# The impact of long-term orientation on compulsive buying behavior: A cross-cultural study

**DOI:** 10.3389/fpsyg.2022.979908

**Published:** 2022-10-12

**Authors:** Pei Wang, Yuqing Zhai

**Affiliations:** ^1^School of Communication, Florida State University, Tallahassee, FL, United States; ^2^School of Business and Tourism Management, Yunnan University, Kunming City, China

**Keywords:** compulsive buying, long-term orientation, money attitude, materialism value, cross-cultural study

## Abstract

The overall purpose of this study was to investigate the wider impacts of cultural values on consumer compulsive buying from a cross-cultural perspective. This study considers the long-term orientation as an extended antecedent to explore the moderating role of materialism value and money attitude on compulsive buying behavior in different cultures. Survey results from 313 Chinese and 309 U.S. consumers indicate that the higher materialistic values drive compulsive buying though some differences exist between consumers in both countries. To specify, American buyers had a higher materialistic orientation and higher compulsive buying tendencies than Chinese consumers. Furthermore, the results indicate that money attitudes are negatively related to compulsive buying behaviors among two countries' consumers. Lastly, this study found that long-term orientations were found to significantly influence money attitudes and compulsive buying among Chinese consumers.

## Introduction

Compulsive buying behavior (CBB) has long been considered the “dark side of consumption” phenomena among psychologists, psychiatrists, and consumer researchers (Moschis, 2017). These assumptions are due to the potentially severe consequences for both consumers and society, such as being more materialistic (Rook, 1987; Mukhtar et al., 2021), feelings of guilt post-purchase (Faber et al., 1987), and financial problems (Dittmar, 2005a,b; Lo and Harvey, 2011). Furthermore, CBB entails consumers' tendency to engage in uncontrollable buying (Flight et al., 2012) and is associated with negative motivations and emotions (i.e., heavy stress, depression, anxiety, and low self-esteem) (Verplanken and Herabadi, 2001; Sun et al., 2004; Moon and Attiq, 2018). In addition, CBB usually lead to bad consequences, such as financial problems, unmanageable debt, post-purchase dissatisfaction, guilt, and family problems (Faber et al., 1987; Dittmar, 2005a,b; Lo and Harvey, 2011).

The past literature examines the impact of psychological factors and personal emotions on compulsive buying behavior. Consumers with lower self-esteem showed greater tendencies have compulsive consumption (O'Guinn and Faber, 1989; Scherhorn et al., 1990; Moon and Attiq, 2018). In addition, when consumers are experiencing negative moods, such as depression (Sneath et al., 2009; Moon and Attiq, 2018), stress (Roberts and Roberts, 2012; Moon and Attiq, 2018), and anxiety (O'Guinn and Faber, 1989; Moon and Attiq, 2018), which often cause compulsive purchases. However, there is a scarcity of studies that discussed the impact of the cultural value of long-term orientations on compulsive buying behavior. With the increasing globalization of the marketplace, scholars have long recognized the importance of cultural characteristics in the context of consumer behavior. Thus, understanding the impact that cultural dimensions and personal values have on compulsive online buying is essential in predicting compulsive buying behavior. To address these gaps in the literature, this study aims at exploring the impact that the cultural value of long-term orientation, money attitude, and materialistic value play on customers' compulsive buying behavior.

The structure of this article is as follows. First, the literature on CBB briefly is reviewed. Next, Hofstede's cultural theory and long-term orientation dimension is presented. After that, hypotheses concerning the relationships between CBB and each values are presented. The relationships are tested with regression analysis using data obtained from a representative sample of Chinses and American consumers. We focused on Chinese and American consumers because prior research indicated that these countries represent two distinct cultures, with scores of variations across six dimensions (Hofstede et al., 2005). After presenting the research findings, we discuss the theoretical and practical implications of the research.

## Conceptual background and hypothesis development

### Compulsive buying behavior

In the marketing context, CBB was defined as “a type of consumer behavior which is inappropriate, typically excessive, and disruptive to the lives of individuals who appear impulsively driven to consume (Faber et al., 1987, p. 134).” In addition, Faber and O'Guinn (1988) claimed CBB was “a response of consumer to an uncontrollable drive or desire to obtain, use, or experience a feeling, substance, or activity that leads to repetitively engage in a behavior that ultimately causes harm to the individual and/or to others” (p. 148). CBB was also a “chronic, repetitive purchasing behavior that occurs as a response to negative events or feelings” (O'Guinn and Faber, 1989, p.149).

A prior study suggested two major groups of factors that may influence CB (Valence et al., 1988). The two factors are (1) psychological factors and (2) socio-cultural factors. To illustrate, psychological factors include personality traits, such as lower self-esteem (O'Guinn and Faber, 1989; Scherhorn et al., 1990; Moon and Attiq, 2018), the ability to fantasize (Bergler, 1958; Feldman and MacCulloch, 1970; O'Guinn and Faber, 1989; Sánchez-Bernardos and Avia, 2004), depression degree (Sneath et al., 2009; Moon and Attiq, 2018), stressful situations (DeSarbo and Edwards, 1996; Zheng et al., 2020), and anxiety (Roberts and Roberts, 2012; Moon and Attiq, 2018), personal values, such as materialistic values (Mowen and Spears, 1999; Dittmar and Drury, 2000; Dittmar, 2005a,b; Faber and O'Guinn, 2008) and hedonic values (Tarka et al., 2022a,b).

Socio-cultural factors include retailing environments, such as salesperson's communication (O'Guinn and Faber, 1989; Hoyer and MacInnis, 2007), sales promotion (Roberts and Pirog, 2004; Rajagopal, 2008), in-store advertising activities (Roberts and Jones, 2001; Roberts and Pirog, 2004), and cultural factors (i.e., Shoham and Segev, 2015; Maccarrone-Eaglen, 2018; Koh et al., 2020). As for demographic factors, the general agreement in the literature is that women are more likely to be compulsive buyers (Shoham and Brencic, 2003; Dittmar, 2005b). The average age of compulsive buyers is lower than normal buyers based on evidence from different countries. This statement was corroborated in France (Lejoyeux et al., 1997), Germany (Scherhorn et al., 1990), and the United States (O'Guinn and Faber, 1989; Hanley and Wilhelm, 1992). In relation to the economic level of countries, prior research has inconsistent conclusions that different development levels lead to consumers' compulsivity (Guo and Cai, 2011; Shoham and Segev, 2015).

### Cultural differences

A cross-culture comparison in consumer behavior is increasingly essential for the online business world. According to Hofstede's cultural indexes, the level of long-term orientation is high in China (score: 118) and low in the United States (score: 29) (Hofstede, 2001). Consistently, the literature claims that significant differences can be found between Chinese and American consumers (Sood and Nasu, 1995; Mazaheri et al., 2011). Similarly, it has been claimed consumers with different cultures would be motivated by various values whenever in the online or the in-store decision-making process (i.e., Mazaheri et al., 2011). There are few cross-cultural studies that discuss the impact of long-term orientation on compulsive buying. Many studies tested individualism and collectivism. Long-term and short-term ordination, as the fifth dimension, which is in Hofstede's culture theory (Frankel et al., 1991), should have in-depth cross-cultural research on this dimension. Furthermore, Chinese consumers rate higher in restrain and long-term orientation. Considering their differences, a question is raised in this study:

**RQ1**: Under the impact of long-term orientation, is the American consumers compulsive buying behaviors different from Chinese consumers?

### CBB and long-term orientation

Hofstede's culture theory is the most widely used model of cultural differences in cross-cultural research (Nardon and Steers, 2009). Long-term orientation (LTO), as the fifth dimension of Hofstede's culture theory, stands for “LTO stands for the fostering of virtues oriented toward future rewards, in particular perseverance and thrift” (Hofstede, 2001, p. 359). LTO also refers to a positive, dynamic, and future-oriented culture (Hofstede, 1991). LTO is a trait that captures how much a person is focused on future gains (Bearden et al., 2006). Long-term-oriented individuals value planning, hard work for future benefit, and perseverance (Bearden et al., 2006).

This dimension is the cultural value of viewing time holistically. People in high LTO levels of society value both the past and the future. They are more flexible toward change and value relationships. They tend to learn from experience and focus on long-term goals. Whereas, people with high short-term orientation (STO) levels, believed actions are important only for their effects in the here and now or the short term. They respect for tradition, focus, and dependence on the past and emphasize the stability of societies. People from STO resist change; thus, they have less room for learning and progress compared to long-term-oriented members.

There is a lack of literature on LTO and compulsive buying. Yet, literature indicated that long-term-oriented people have a high level of self-control and plan for their future goals (Bearden et al., 2006; Nepomuceno and Laroche, 2017). People with LTO are most likely to buy products that are an actual or utilitarian need (Joshanloo and Park, 2021). However, compulsive buyers are intensely focused on adventure, sensation seeking, emotional excitement, or the hedonic goal of shopping itself (Ali, 2020). In a compulsive shopping process, hedonic shopping experiences are remedies for negative feelings (O'Guinn and Faber, 1989). That said, compulsive buyers need entertainment or seek some fun to deal with their negative emotions. They may focus more on present emotions and be willing to cope with them shortly. Thus, compulsive buyers most likely have a low level of self-control and a low level of long-term orientation due to their hedonism. Based on these assumptions, a negative association between LTO and CBB is expected. The following hypothesis is proposed:

**H1:** Long-term orientation (LTO) will be negatively associated with compulsive buying behavior (CBB).

### CBB and materialistic value

Materialism value is a widely tested culture value in compulsive buying studies. Previous research suggested that materialism is a possible predictor of compulsive buying (O'Guinn and Faber, 1989; Mowen and Spears, 1999; Dittmar and Drury, 2000; Dittmar, 2005a,b; Faber and O'Guinn, 2008). Dittmar (2005a,b) also found support that materialistic value endorsement is the strongest predictor of compulsive buying. Materialistic value refers to “the importance a person places on material possessions and their acquisition, as a necessary or desirable form of conduct to reach desired end states” (Richins and Dawson, 1992; p. 307). It refers to a “set of centrally held beliefs about the importance of “material” possessions in one's life (Richins and Dawson, 1992, p. 308).” The literature revealed that compulsive buyers had a higher materialistic orientation (O'Guinn and Faber, 1989; Mowen and Spears, 1999), perceived materialistic consumption as an “aid” in repairing one's mood, and facilitated the development of one's self-identity (Dittmar and Drury, 2000).

Materialistic individuals, like compulsive buyers, possess low self-esteem, report a poor quality of life, and are generally dissatisfied with life (Richins and Dawson, 1992; Sirgy, 1998; Roberts and Clement, 2007). Prior studies suggested that material possessions serve as surrogates for inadequate or non-satisfying interpersonal relationships; more materialistic individuals place a higher emphasis on possessions than on interpersonal relationships (Belk, 1985; Richins, 1994; Dittmar, 2005a,b; Roberts et al., 2003). When individuals' needs for safety and security are not met, they tend to emphasize desires and materialistic values, which translate into buying as a mechanism to climb up or claim status (Roberts and Sepulveda, 1999; Neuner et al., 2005; Roberts et al., 2008). Perhaps because of this worldview, materialists often look for excitement, self-fulfillment, fun, and enjoyment (Richins and Dawson, 1992); thus, materialists are more likely to engage in compulsive shopping. So, the hypothesis is stated as an assumption:

**H2:** Materialistic value will be positively associated with compulsive buying behavior (CBB).

Many similar studies indicated that compulsive consumers have high materialistic tendencies than non-compulsive buyers (Dittmar, 2005a,b; Eren et al., 2012). Previous studies examined the effect of materialism and long-term orientation on consumer ethics (Arli and Tjiptono, 2014) and consumers' self-efficacy (Salim et al., 2020). However, in these studies, materialism value and LTO are both independent variables. There are limited studies that examine the relations between LTO to materialism value, which is a significant gap of the literature. As one of six core cultural values, long-term orientation may somewhat affect the materialism value on compulsive buying behaviors. Thus, the following relationship is proposed.

**H3:** Long-term orientation (LTO) will mediate the relationship between materialistic value and compulsive buying behavior (CBB).

### CBB and money attitude

Several studies found that money attitude can shape consumers' values and then affect their behaviors. Money attitude is a multidimensional construct, and individuals associate different symbolic meanings with money (Medina et al., 1996). The measurement of money attitude includes three dimensions: power prestige, retention time, and quality (Gresham and Fontenot, 1989; Medina et al., 1996). The retention time dimension presents that individuals used the money to plan and prepare for the future (Yamauchi and Templer, 1982). Previous research found that the retention-time dimension significantly impacted consumers' compulsive buying (Roberts et al., 1999; Li et al., 2009). Thus, the present study predicted a negative association between money attitude and compulsive buying,

**H4:** Money attitude will be negatively associated with compulsive buying behavior (CBB).

Therefore, individuals with a high score of the retention time dimension are more careful about financial planning. According to this statement, LTO may somewhat affect the retention time dimension of money attitude. Thus, the following association is proposed.

**H5:** LTO is significantly associated with money attitude.

**Figure 1:**
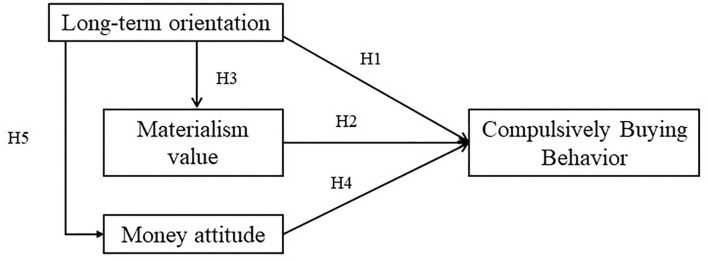
Conceptual model.

In sum, this study predicts that LTO, as an extended antecedent, will affect materialism value and money attitudes. Then, LTO, materialism values, and money attitudes will impact on compulsive buying behavior. See Figure 1 for the conceptual model.

## Research design and methodology

### Research design

The research design for this study is a cross-sectional study design, which is a one-time measurement of exposure and outcome (Setia, 2016). The data collection process for both countries was conducted for 4 months and started at the same time to keep the collection time consistent. Furthermore, due to the unique impact of different cultures on consumers, scholars need to use objective and consistent scales to explain and predict consumers' values and behaviors (Gudykunst, 2003). An online survey is the most common method in cross-cultural studies for its methodological advantages. Thus, this study primarily used the online survey method to test the hypotheses and examine the research questions.

This study used several statistical tools to achieve the data analysis goals. First, factor analysis will be run to test item loadings for assessing convergent and discriminant validity. In addition, average variance extracted (AVE) and composite reliabilities (CR) will be calculated to verify the data have sufficient levels of convergent validity among the reflective constructs. Then, to validate the instrument and gain internal validation for the study, Cronbach tests were run across constructs. It is an approach that calculates reliability based on the correlation between multiple items used to measure a single concept. Next, mean calculation and standard deviation (SD) calculation will be used to get mean values and SD values for each variable. Last, to test the proposed hypotheses (H1–H5) and answer the research question (R1), a series of regression analyses will be conducted.

### Sample and data collection

An online survey created in English and Mandarin was administered using convenience samples through social media. The advantages of the online survey method are clear: It can provide easy access to a greater number of people (Merrigan and Huston, 2020), which is helpful for cross-cultural researchers to post the survey. Second, the anonymity and confidentiality of the survey may eliminate participants' privacy and safety concerns (Merrigan and Huston, 2020). Next, participants may be more honest in answering the study than doing an interview (Merrigan and Huston, 2020). Other advantages of using online surveys include increased reach, high speed of survey return, low cost, anonymity, and decreased bias from the interviewer (Sheehan and Hoy, 1999).

For China, the data will be collected by using Wenjuanxing (WJX, https://www.wjx.cn/), which is a Chinese commercial online survey service provider. For Chinses survey, the questionnaire and all measures were translated into Mandarin following the translation-back-translation technique. For the United States, the data will be established by Qualtrics and will be collected by using Amazon Mechanical Turk (M-Turk, https://www.mturk.com/).

A total of 705 questionnaires were distributed, which includes 355 Chinese surveys and 350 US surveys. A total of 628 questionnaires were returned, and the whole useable rate is 82.4%. A total of 622 available samples were collected from March to June 2022. Both surveys were collected at the same time to keep the time consistent. The sample was composed of 309 Americans and 313 Chinese participants. As a whole, more than half of the respondents are between the ages of 20 and 39 (57.7%). The sample achieved a gender-balanced sample (304 males and 318 females). Education was distributed normally (25% had a high school education or less, 56.9% had some college degree or currently in college, and 18% had bachelor's degree or post-graduate degree). Furthermore, 64.6% of participants were employed full-time (see Table 1).

**Table 1:** China sample and the US samples.

	**China sample** ***N* = 313**	**Percent (%)**	**The US sample** ***N* = 309**	**Percent (%)**
**Gender**				
Female	149	47.6	169	54.7
Male	164	52.4	140	45.3
**Age**				
20–29	128	40.9	98	31.7
30–39	54	17.3	79	25.6
40–49	71	22.7	64	20.7
50–59	44	14.1	38	12.3
60+	16	5	30	9.7
**Education**				
High school of less	87	27.8	69	22.3
Some colleges or currently in college	167	53.4	187	60.5
College degree or post-graduate	59	18.8	53	17.2
**Occupation**				
Working professional	195	62.3	207	67
College student	92	29.4	87	28.1
Housewife or unemployed	26	8.3	18	4.9

### Measurement

The instrument used in this study included constructs already created and validated in English. To measure the variables proposed in the conceptual model, constructs will be adapted and translated to English. Then, as previously addressed, the survey will be back-translated to English for validation testing. Long-term orientation will be measured using the Bearden et al. (2006) eight-item scale. The materialistic value will be tested using Richins (2004) 15-item materialistic value attitude scale (MVS). The 18-item money attitudes scale (MAS) includes three factors: power prestige (eight items), retention time (six items), and quality (four items) (Gresham and Fontenot, 1989; Medina et al., 1996). The compulsive buying tendency is measured using a 13-item scale from Edwards (1993). This scale is based on the scale of Faber and O'Guinn (1988), to determine the individuals' compulsive degree in their buying behavior.

There are 54 items in total in the questionnaire for measuring all the constructs. All the variables will use a seven-point Likert scale in the instrument (1 for strongly disagree, 2 for disagree, 3 for somewhat disagree, 4 for neither agree nor disagree, 5 for somewhat agree, 6 for agree, and 7 for strongly agree). Furthermore, the demographic questions were included as well. For example, the questionnaire includes age, gender, education level, occupation, income, and ethnicity.

## Data analysis and results

### Measure validation

Before evaluating the structural model, we ensured the validity and reliability of the data. To this end, we used the Statistical Package for the Social Science (SPSS) 24.0 tool. Fifty-four items relating to all the scales were factor analyzed in each scale, using principal component analysis with varimax rotation. Following the Fornell and Larcker (1981) criteria, the loadings of the individual items must be at least 0.7 for the data to be considered valid. The analysis showed two items from the money attitude scale, four items from the materialistic attitude scale, and two items from the compulsive buying scale did not meet this criterion. Therefore, these items were removed from the respective scales. The results in Table 2 show standardized factor loadings and commonalities for each construct.

**Table 2:** Survey items and their loadings and commonalities.

**Item**	**Factor loadings**	**Commonalities**	**Item**	**Factor loadings**	**Commonalities**
LTO-1	0.86	0.67	MAS-RT1	0.91	0.69
LTO-2	0.88	0.62	MAS-RT2	0.71	0.55
LTO-3	0.88	0.60	MAS-RT3	0.95	0.53
LTO-4	0.87	0.58	MAS-RT4	0.94	0.64
LTO-5	0.88	0.60	MAS-RT5	0.90	0.62
LTO-6	0.90	0.63	MAS-RT6	0.79	0.63
LTO-7	0.81	0.67	MAS-Q1	0.94	0.54
LTO-8	0.89	0.58	MAS-Q2	0.76	0.67
MVS-1	0.80	0.68	MAS-Q3	0.92	0.50
MVS-2	0.90	0.68	MAS-Q4	0.80	0.58
MVS-4	0.80	0.64	CB-1	0.78	0.55
MVS-5	0.83	0.62	CB-4	0.77	0.69
MVS-8	0.95	0.63	CB-5	0.71	0.60
MVS-9	0.80	0.59	CB-6	0.71	0.57
MVS-10	0.85	0.60	CB-7	0.95	0.57
MVS-11	0.86	0.65	CB-8	0.87	0.65
MVS-12	0.79	0.54	CB-9	0.75	0.50
MVS-14	0.93	0.60	CB-10	0.74	0.56
MVS-15	0.78	0.66	CB-11	0.85	0.67
MAS-PP1	0.83	0.63	CB-12	0.89	0.69
MAS-PP2	0.75	0.59	CB-13	0.72	0.65
MAS-PP5	0.77	0.60			
MAS-PP6	0.88	0.57			
MAS-PP7	0.94	0.65			
MAS-PP8	0.84	0.51			

LTO, long-term orientation; MVS, materialistic value scale; MAS, money attitude scale; CB, compulsive buying.

Next, Cronbach's alphas will be run to test the reliability of factor constructed. Cronbach's alpha is the common measure of internal consistency (or interitem reliability). It is an approach that calculates reliability based on the correlation between multiple items used to measure a single concept (Chambliss and Schutt, 2018). Overall, the results revealed that the reliability levels were above the recommended level of 0.7 (Nunnally and Bernstein, 1994).

Furthermore, convergent and discriminant validity will be tested. To verify the data have sufficient levels of convergent validity among the reflective constructs, we need to test the average variance extracted (AVE) and composite reliabilities (CR). According to the definition from Fornell and Larcker (1981), the results show that AVE is >0.5, and CR is >0.7, which satisfies the criteria.

Taken together, the results indicate that the proposed model appears to fit the data from two countries. Tables 3, 4 present the Cronbach's alphas, means, standard deviations, AVE, and CR.

**Table 3:** Scale reliability levels (α), means, standard deviations (SD), average variance extracted (AVE), and composite reliabilities (CR) (China sample).

**Variable**	**Alpha**	**Means (SD)**	**AVE**	**CR**
LTO	0.86	4.63 (0.84)	0.84	0.56
MV	0.78	3.84 (0.75)	0.84	0.51
MA	0.84	4.32 (0.82)	0.91	0.54
CBB	0.79	3.77 (0.90)	0.84	0.61

**Table 4:** Scale reliability levels (α), means, standard deviations (SD), average variance extracted (AVE), and composite reliabilities (CR) (The US sample).

**Variable**	**Alpha**	**Means (SD)**	**AVE**	**CR**
LTO	0.79	4.23 (0.86)	0.82	0.53
MV	0.93	4.12 (0.94)	0.78	0.52
MA	0.80	4.34 (0.81)	0.89	0.57
CBB	0.74	3.98 (0.77)	0.79	0.59

### Model testing and multigroup comparison

**Figure 2:**
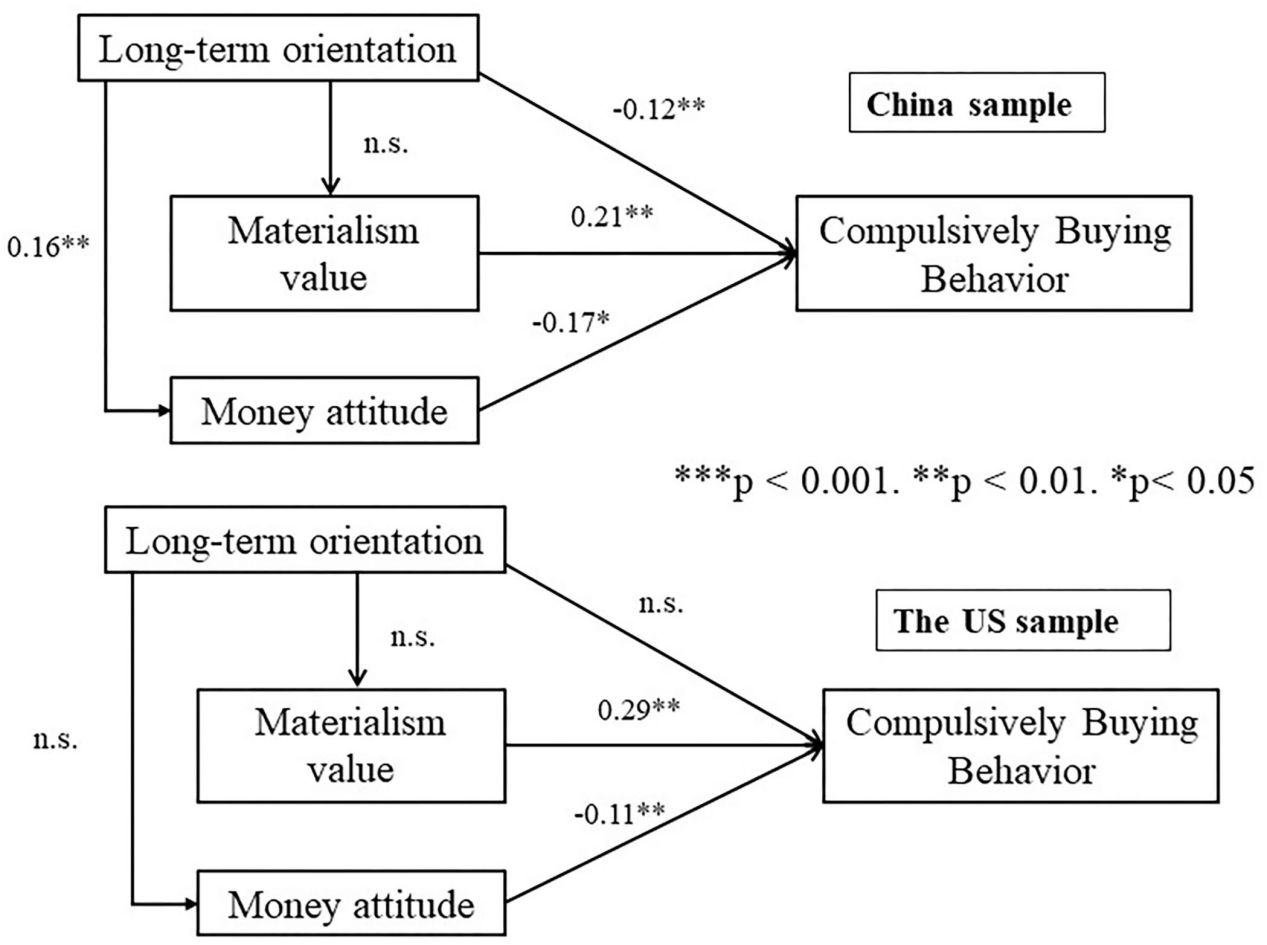
Multigroup regression weights in the conceptual model. *p < 0.05, **p < 0.01, ***p < 0.001, n.s. means no significant relation.

After ensuring sufficient validity and reliability of the data, we tested the proposed research model by using regression analysis. Figure 2 shows the results. The standard coefficients indicate that, among Chinese consumers, the impact of long-term orientation (LTO) on materialistic value (H3, ß = −0.063, *p* = 0.389) is not statistically significant, whereas, on American consumers, the influence of LTO on compulsive buying behaviors (CBB) (H1, ß = −0.076, *p* = 0.254), the impact of LTO on materialistic value (H3, ß = −0.044, *p* = 0.47), and the impact of LTO on money attitude (H5, ß = −0.054, *p* = 0.53) are not significantly.

Furthermore, the regression weights demonstrate that materialistic value has a significant positive effect on CBB among Chinese (ß = 0.21, *p* < 0.01) and Americans (ß = 0.29, *p* < 0.01), which indicates H2 is supported. Money attitude significantly negatively affects CBB among Chinese consumers (ß = −0.17, *p* < 0.05) and Americans buyers (ß = −0.11, *p* < 0.01). Thus, H4 is supported. Overall, H2 and H4 are supported among both Chinses and American samples.

In addition, for Chinese consumers, LTO shows a significant negative impact on CBB (ß = −0.12, *p* < 0.01); LTO positively influences money attitude significantly (ß = 0.16, *p* < 0.01). Therefore, H1 and H5 are supported in China. See the summarizing results in Figure 2.

## Conclusion and discussion

This study examined the relationships among personal value (i.e., materialistic value), personal attitude (i.e., money attitude), and cultural dimensions (i.e., long-term orientation) with consumer compulsive buying among China and the U.S. adults. In addition, it also tested the relationship between long-term orientation with personal value and personal attitude. By doing so, we attempt to address existing gaps in the literature with respect to three areas: (1) the lack of a proposed model that examines long-term orientation as an antecedent value on materialistic value and money attitude, and then the impact on compulsive buying behavior; (2) the need to extend the testing of the antecedents of culture dimensions beyond collectivism/individualism and power distance, and (3) the limited research that studies countries other than the United States.

In addition, this study presents theoretical contributions to current studies. The result of the multigroup analysis shows that materialistic value leads to compulsive buying behavior in two countries (0.21 in China; 0.29 in the United States). It confirms the findings from previous research on compulsive purchases. The previous study indicated that materialistic value is the strongest predictor of compulsive buying (Dittmar, 2005a,b). Comparing the regression weight of materialistic value to compulsive buying behavior, an important question is raised up. Why is the regression weight of materialistic value in the US higher than Chinese consumers? Materialistic individuals place a higher emphasis on possessions than on interpersonal relationships, that said, they concentrate on themselves more than on the care of others. In addition, according to Hofstede's culture dimension score, the individualism level of American consumers is higher than chinses consumers, which indicated that American consumers largely express their self-concept when they shop. They take more care of themselves and their possessions. That is why American buyers had a higher materialistic orientation (3.84 in China, 4.12 in the United States) and a higher compulsive buying tendency than Chinese consumers (3.77 in China, 3.98 in the United States).

Furthermore, the results show that money attitude is negatively related to compulsive buying behaviors among two countries' consumers (−0.17 in China; 0.11 in the United States). This finding confirms the previous study and explores the relations between money attitude and compulsive buying in China and the United States. Previous findings suggest that money attitudes are closely related to compulsive buying (Roberts and Jones, 2001); this study investigated the relations and found that money attitudes are negatively associated the compulsive buying. In addition, the study found that the relation coefficients of money attitude and compulsive buying in US consumers are higher than in Chinese consumers. This finding is another explanation for the antecedent factors of compulsive buying behaviors in two countries. Money attitude, as one of the consumption values, significantly influences the consumers' compulsive buying behaviors.

Another important finding is that Chinese participants more plan for the long-term than American participants (4.63 in China, 4.23 in the United States). This study tested the LTO value among two country consumers at the individual level, and the result verified the differences in scores within the two countries from Hofstede's long-term orientation dimension (Hofstede et al., 2005). In addition, these differences further demonstrated the relevant studies (Bearden et al., 2006; Nepomuceno and Laroche, 2017) that long-term-oriented people have a high level of self-control and plan for their future goals (Bearden et al., 2006; Nepomuceno and Laroche, 2017). People with long-term orientation are most likely to buy products that are an actual or utilitarian need (Joshanloo and Park, 2021). Thus, people with a high level of LTO may have less tendency on compulsive buying.

## Practical implications

In marketing practice, several implications can be tied to these results. By understanding long-term orientation's role in different cultures, marketers can use its predictive power to tailor their marketing approaches to highlight specific cultural marketing strategies. For example, the segments that are long-term-oriented may be more inclined to purchase the functional and valuable product at a lower cost. In addition, the segments may have lower motivations for compulsive buying. In contrast, cultures that exhibit a more significant amount of short-term orientation will opt for the latest technological gadgets and pay for more outstanding using experience (de Mooij, 2015).

## Limitations and future research

Despite the theoretical contributions and practical implications of this study, there are several limitations. First, the data were collected online using different convenience sample techniques. Even though the age and gender group distribution are almost balanced, the sampling techniques with quotas to collect samples should be similar in a cross-culture study. In addition, this study did not figure out the shopping environment in the survey, whether online shopping environment or offline shopping, and future research should test the model in a different environment (i.e., offline retailing and online live-video shopping) and limited to one specific context. Besides, this study was limited to two countries. Further study can consider replicating the model in cultures with a different level of long-term orientation, such as Mexico, Italy, or India, which can be used to compare the effect of long-term orientation vs. short-term-oriented cultures. Finally, future research should explore other personal values and cultural values in the proposed model, such as indulgent (Hofstede's sixth cultural dimension), hedonic values, utilitarian values, and personal emotions.

## Data availability statement

The original contributions presented in the study are included in the article/supplementary material, further inquiries can be directed to the corresponding author.

## Author contributions

PW: reviewing the literature and writing manuscript. YZ: collecting data and translating survey. All authors contributed to the article and approved the submitted version.

## Conflict of interest

The authors declare that the research was conducted in the absence of any commercial or financial relationships that could be construed as a potential conflict of interest.

## Publisher's note

All claims expressed in this article are solely those of the authors and do not necessarily represent those of their affiliated organizations, or those of the publisher, the editors and the reviewers. Any product that may be evaluated in this article, or claim that may be made by its manufacturer, is not guaranteed or endorsed by the publisher.
